# Complete mitochondrial genome of critically endangered *Crocidura nicobarica* (Soricidae: Eulipotyphla) from the Great Nicobar Island, India

**DOI:** 10.1080/23802359.2021.1999188

**Published:** 2021-11-18

**Authors:** Shantanu Kundu, Manokaran Kamalakannan, Kaomud Tyagi, Vikas Kumar

**Affiliations:** a Molecular Systematics Division, Centre for DNA Taxonomy, Zoological Survey of India, Kolkata, India; b Mammal and Osteology Section, Zoological Survey of India, Kolkata, India

**Keywords:** Shrew, AN archipelago, endemic species, mitogenome, phylogeny

## Abstract

The mitogenome (17,388 bp) of the Nicobar shrew, *Crocidura nicobarica* was determined in the present study. The mitogenome comprises 13 PCGs (11,427 bp), 22 tRNAs (1507 bp), two rRNAs (2538 bp), and a major non-coding control region (1932 bp). The Maximum Likelihood phylogeny clearly discriminates all the studied *Crocidura* species with high bootstrap support by concatenated PCGs. The studied species, *C. nicobarica* shows a close relationship with *Crocidura orientalis*, distributed in Java, Indonesia. The lineage diversification and zoogeographic patterns are congruent in the present analyses and encouraged further sampling and more molecular data to elucidate their in-depth evolutionary relationship.

## Introduction

The *Crocidura* is a species-rich genus with 199 species widely distributed in Africa, Europe, and Asia (Burgin et al. 2018; Wilson and Mittermeier 2018). These terrestrial and insectivorous mammalian species are commonly known as white-toothed shrews (Lekagul and McNeely 1977). The extant *Crocidura* species can be easily distinguished by their small to medium-sized body with typically short dense gray fur, a large first-unicuspid tooth, protrudes forward and hooked, a small cusp exist behind the main cusp, unpigmented teeth, and absence of zygomatic arches (Martin et al. 2011). A recent study reported that a total of 12 *Crocidura* species are known from India with a new discovery of *Crocidura narcondamica* from Andaman and Nicobar (AN) Archipelago (Kamalakannan et al. 2021). Among them, five species viz., *C. andamanensis* (Critically Endangered), *C. hispida* (Vulnerable), *C. jenkinsi* (Critically Endangered), *C. narcondamica* (Not Evaluated), and *C. nicobarica* (Critically Endangered) are endemic to the AN Archipelago (IUCN 2021).

The natural history and molecular information of *Crocidura* species from the AN Archipelago is poorly known except for a few studies (Molur et al. 2005; Kundu et al. 2020; Kamalakannan et al. 2021). The molecular studies were rigorously utilized to elucidate the phylogenetic relationship and lineage diversification of *Crocidura* species throughout the world (Dubey et al. 2008; Esselstyn et al. 2009; He et al. 2010; Giarla and Esselstyn 2015; Stanley et al. 2015; Demos et al. 2016). Further, the complete mitochondrial genome-based approach is also evidenced to be successful for systematics and evolutionary research of a wide group of taxa including mammals (Arnason et al. 2002; Pacheco et al. 2011; Finstermeier et al. 2013; Kundu et al. 2018; Kundu et al. 2020). So far, the complete mitogenome of 16 *Crocidura* species were generated from different geographical regions. However, the mitochondrial genome information of *C. nicobarica* is not available in the global GenBank database. To fill the gap of knowledge, the present study aimed to determine the complete mitogenome of *C. nicobarica* from the Great Nicobar Island, India. The phylogenetic analyses were estimated to infer the evolutionary relationship of *C. nicobarica* comparing with other *Crocidura* species.

## Materials and methods

A fresh natural kill shrew specimen was collected from the Galathea, Great Nicobar Island (6.87 N 93.88E). The experimental procedures were approved by the Zoological Survey of India (ZSI) and were carried out in accordance with relevant guidelines in compliance with ARRIVE 2.0. guidelines (https://www. arriveguidelines.org) (Percie du Sert et al. 2020). The muscle tissue sample was aseptically collected from the individual and stored in 70% ethanol for downstream molecular analyses and vouchered at the Mammal and Osteology section, ZSI, Kolkata under voucher ID (Reg. No. 28533). The species was morphologically identified as *C. nicobarica* by their external morphological characters, compared with other National Zoological Collections of ZSI and based on the published information (Miller 1902). The studied individual has bristly sooty brown dorsal fur with a slender tail, head and body length is 120 mm, and tail length is 90 mm.

The tissue sample was homogenized with 1 ml buffer containing 0.32 M Sucrose, 1 mM EDTA, and 10 mM TrisHCl by the WiseTis HG-15 homogenizer. The working solution was centrifuged at 700 g for 5 min at 4 °C to remove the nuclei and cell debris. The supernatant was collected in 1.5 ml centrifuge tube and centrifuged at 12,000 g for 10 min at 4 °C to precipitate the mitochondrial pellet.The pellet was re-suspended in 500 µl of buffer (50 mM TrisHCl, 25 mM of EDTA, 150 mM NaCl) and incubated at 56 °C for 1–2 h along with 20 µl of proteinase K (20 mg/ml). The mitochondrial DNA was extracted by using QIAamp DNA Investigator Kit (QIAGEN Inc.).

The complete mitogenome sequence and assembly were carried out at PHIXGEN Pvt. Ltd. Gurugram, India (http://www.phixgen.com). The mitochondrial DNA (>100 ng) was used in Illumina TruSeq Nano DNA HT library preparation kit for library assembly (Illumina, Inc, USA). Total >4 million raw reads were generated through Illumina NextSeq500 (150 × 2 chemistry) after obtaining the expected concentration (650.25 pg/µl) and mean peak size (465 bp) (Illumina, Inc, USA). The high-quality reads were downsampled to 2 million using Seqtk (https://github.com/lh3/seqtk) and assembled using NOVOPlasty v2.6.7 using default parameters (Dierckxsens et al. 2017).

The typical circular representation of the generated mitogenome of *C. nicobarica* was plotted by CGView Server (http://stoth ard.afns.ualbe rta.ca/cgview_server/) with default parameters (Grant and Stothard 2008). Further, the contig was subjected to confirmation by the MITOS v806 online webserver (http://mitos.bioinf.uni-leipzig.de). The direction and arrangements of protein-coding genes (PCGs), transfer RNA (tRNA), and ribosomal RNA (rRNA) were confirmed through MITOS online server (http://mitos.bioinf.uni-leipzig.de) (Bernt et al. 2013). The mitogenome was submitted to the GenBank database using the NCBI Bankit submission tool.

On the basis of taxonomic classification, 14 *Crocidura* species mitogenomes were downloaded from GenBank to construct the phylogenetic dataset (Table S1). The genome sizes and nucleotide compositions of all the studied species were calculated using MEGA X (Kumar et al. 2018). The overlapping regions and intergenic spacers of *C. nicobarica* mitogenomes were calculated manually by Microsoft Excel spreadsheet. The PCGs dataset (11,352 bp) were aligned and concatenated using TranslatorX (with MAFFT algorithm with L-INS-i strategy and GBlocks parameters) and SequenceMatrix v1.7.84537 (Abascal et al. 2010; Vaidya et al. 2011). The best fit model (GTR + I + G) was estimated by PartitionFinder 2 using lowest BIC criterion (Lanfear et al. 2017) and the maximum-likelihood (ML) tree was constructed using the IQ-Tree web server with 1000 bootstrap support (Trifinopoulos et al. 2016). The mitogenome of the Asian house shrew, *Suncus murinus* (accession No. NC_024604) was used as an out-group in the dataset.

## Results and discussion

**Figure 1.**
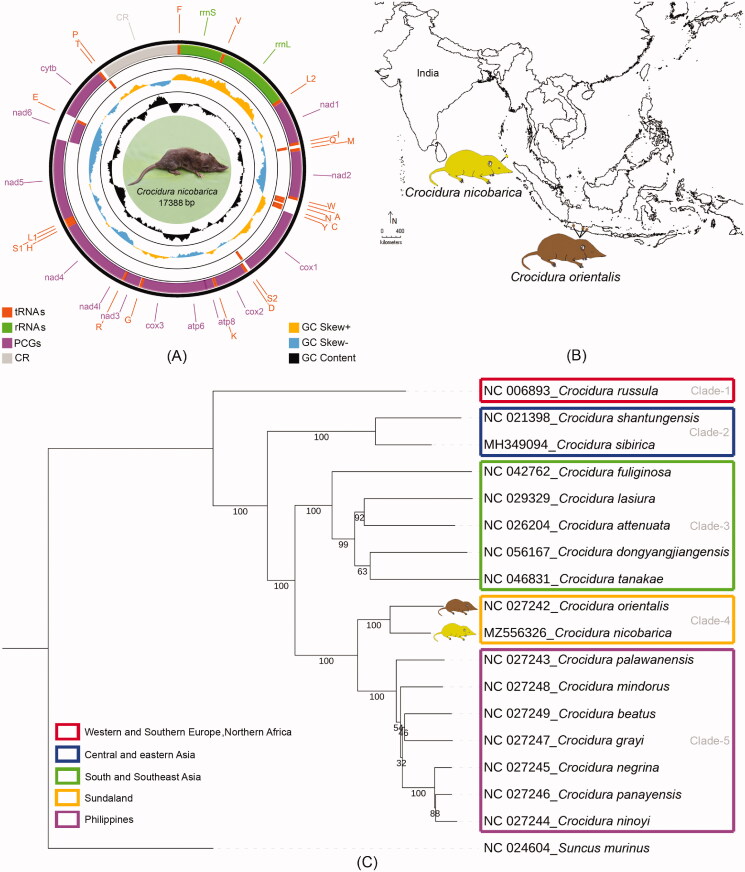
(A) The species photograph and mitochondrial genome of *C. nicobarica*. PCGs are marked by orcid color boxes, rRNAs are marked by green color boxes, tRNAs are marked by red color boxes, and CR is marked by gray color box. tRNAs are encoded according to their single-letter abbreviations. The GC content is plotted using a black sliding window; GC-skew is plotted using orange and blue color sliding windows as deviation from the average of the complete mitogenome. (B) Distribution pattern of *C. nicobarica* and its sister species *C. orientalis* in the Great Nicobar Island, India and Java, Indonesia respectively. (C) The PCGs based ML phylogeny showed distinct clustering of C*. nicobarica* and other *Crocidura* species.

The complete mitochondrial genome of *C. nicobarica* (accession no. MZ556326) was 17,388 bp with 35.4% GC content. The highest mitochondrial genome length was for *Crocidura attenuata* (17,534 bp), while lowest for *Crocidura orientalis* (15,452 bp). This length variation was detected due to the lack of control region in other *Crocidura* species. The *C. nicobarica* mitogenome contained 37 genes, comprising 13 PCGs, 22 tRNAs, 2 rRNAs, and a major non-coding control region (CR). Among them, nine genes (*nad6* and eight tRNAs) were placed on the negative strand, while the remaining 28 genes were placed on the positive strand (Table S2, Figure 1(A)). A total of 15 intergenic spacer regions with a total length of 80 bp were observed in *C. nicobarica* mitogenome with the longest region (37 bp) between tRNA-Asparagine (*trnN*) and tRNA-Cysteine (*trnC*) (Table S2). Further, 12 overlapping regions with a total length of 96 bp were identified in *C. nicobarica* mitogenome. The longest overlapping region (43 bp) was observed between the ATP synthase F0 subunit 8 (*atp8*) and ATP synthase F0 subunit 6 (*atp6*). The total length of PCGs was 11,427 bp in *C. nicobarica*, which represents 65.71% of the complete mitogenome. Most of the PCGs of *C. nicobarica* initiated with an ATG start codon; however, the ATA initiation codon was found in *nad5* and *nad3*, ATT in *nad2*. The TAA termination codon was used by eight PCGs, TAG by *nad2* and *nad3*, AGA by *cytb*, incomplete TA(A) by *cox3* and T(AA) by *nad4* respectively. The total length of ribosomal RNA, transfer RNA and control region was 2538 bp, 1507 bp, and 1932 bp respectively. The GC content of all *Crocidura* mitogenomes was ranging from 34.70% (*C. sibirica*) to 37.30% (*C. rusulla*) in the present dataset. The PCGs based ML phylogeny clearly discriminate all the studied *Crocidura* species with high bootstrap support (Figure 1(C)). The studied species, *C. nicobarica* shows a close relationship with *C. orientalis*, distributed in Java, Indonesia. The present phylogenetic analysis of mitochondrial genomes of representative *Crocidura* species inferred five clades: Clade-1 represents the species distributed in the Western and Southern Europe, Northern Africa; Clade-2 represents the species from the Central and Eastern Asia, Clade-3 from South and Southeast Asia, Clade-4 from Sundaland, and Clade-5 from the Philippines (Figure 1(B)). The present phylogenetic analysis corroborates the earlier hypothesis (Li et al. 2021) and adds another lineage of *Crocidura* species from the Sundaland.

Due to the drift of Indian plate from Africa-Madagascar- Seychelles and toward the Eurasian plate, the AN archipelago constitutes an unparalleled make-up during Miocene–Pliocene (Esselstyn et al. 2009; Upham et al. 2019). This plate tectonic also impelled multiple chances for animal oversea dispersal and biological connections between South and Southeast Asian countries (Kamalakannan et al. 2021). Nevertheless, the bathymetric study also evidenced a prominent connection between the AN archipelago to Sundaic Island by the well-developed seamounts and volcanic arc-chain (Rodolfo 1969; Tripathi et al. 2017). The present phylogeny of *Crocidura* species showed congruent output with the zoogeographic pattern of the Indo-Malayan and Sundaic realms (Esselstyn & Brown 2009). We also presumed that the South China Sea might have created a biogeographic barrier for the dispersal of *Crocidura* species within South and Southeast Asian countries. Due to the independent colonization events many *Crocidura* species showed an unique dispersal affinity and endemism in the different oceanic islands. To infer this astonishing pattern, we encourage generating more molecular data of this mammalian group from diverse geographical regions to elucidate their in-depth phylogenetic relationships and lineage diversification in South and Southeast Asia.

## Authors’ contributions

Conceptualization: S.K., V.K.; Data curation: K.T., S.K. M.K.; Formal analysis: S.K. M.K.; Funding acquisition: V.K.; Investigation: S.K., K.T.; Methodology: S.K. M.K., K.T.; Project administration: V.K.; Resources: V.K., K.T.; Software: S.K. K.T.; Supervision: V.K.; Validation: S.K. M.K., K.T.; Visualization: S.K., V.K.; Writing – original draft: S.K., M.K., K.T.; Writing – review & editing: S.K., V.K.

## Data Availability

The genome sequence data that support the findings of this study are openly available in GenBank of NCBI at [https://www.ncbi.nlm.nih.gov] (https://www.ncbi.nlm.nih.gov/) under the accession no. MZ556326. The associated BioProject, SRA, and Bio-Sample numbers are PRJNA767501, SRR16122703, and SAMN21906696 respectively.
